# Leveraging the bi-directional links between health and education to promote long-term resilience and equality

**DOI:** 10.1177/01410768211066890

**Published:** 2022-01-06

**Authors:** Anant Jani, Chloe Lowry, Eloise Haylor, Shamila Wanninayake, David Gregson

**Affiliations:** 1 Oxford Martin School, University of Oxford, Oxford OX1 3BD, UK; 2 Heidelberg Institute of Global Health, University of Heidelberg, 69120 Heidelberg, Germany; 3 University College London, London WC1E 6BT, UK; 4 Big Change, London WC2R 0EX, UK; 5 Royal College of General Practitioners, London NW1 2FB, UK; 6 Gregson Family Foundation, West Sussex PO19 1UF, UK; 7 BeeWell: Manchester Wellbeing Programme, Manchester M13 9PL, UK

In the 1970s, a longitudinal study in Barbados set out to explore impacts on children exposed to episodes of malnutrition (protein and calories) in the first year of life. Forty years after their initial exposure, researchers found that, even after adjusting for childhood socioeconomic factors, individuals experiencing severe malnutrition had a variety of negative outcomes. Of these individuals, 15%–60% had deficits in attention and cognition compared to healthy controls throughout childhood, adolescence and adulthood. A substantial proportion also had increased mood and behavioural problems including maladaptive personality traits, cognitive rigidity, slow processing and impaired cognitive control. One in four affected individuals could be characterised as intellectually disabled based on their IQ scores – a prevalence rate nine times higher than the control group.^1^

These startling results demonstrate the long-lasting negative impacts of acute nutritional shocks. Given the range of impacts, especially cognitive, it is easy to see how educational outcomes would also have been negatively affected for these individuals. While this is an extreme case, it is important to acknowledge that there are myriad ways health can affect education. A study in England found that year 7 pupils with mental health difficulties had greater absenteeism and poorer educational attainment.^2^ Additionally, common manifestations of poor health like obesity/overweight, smoking and sleeping disorders have also been shown to negatively impact educational outcomes. More serious illnesses before the age of 21 have been shown to decrease education by 1.4 years on average.^3^ Studies have also found that physical exercise and overall good child health can improve educational outcomes.^3^

These findings point to an essential role for early interventions from the public health and healthcare systems in improving life chances for individuals. It is important to note, however, that interventions in these sectors are necessary but not sufficient. Several longitudinal studies point to a very significant role of the education sector on impacting the long-term health and resilience of individuals and society more generally.

In this commentary, we explore the interlinkages between health and education as well as the intergenerational impacts of poor education. We then consider the implications of the COVID-19 pandemic and make suggestions for how we can minimise damage caused by it while also building greater resilience of individuals by leveraging investments in the education sector to improve life chances for individuals. We hope that the insights from this commentary can directly help inform and influence education and health policy.

## Education impacts on health

A recent study analysing nationally representative samples of individuals aged 50+ from 14 Organisation for Economic Cooperation and Development (OECD) countries found interesting results that suggest important links between education and health. The authors found that one additional year of schooling was associated with a 6.85%-point reduction in individuals reporting poor health and a 3.8%-point and 4.6%-point reduction in self-reported difficulties with activities of daily living (activities of daily living) and instrumental ADLs, respectively. Furthermore, educational attainment was associated with a 4.4%-point reduction in the probability of individuals having a chronic disease.^4^ Another study from the European Union (EU) Ageing Trajectories of Health: Longitudinal Opportunities and Synergies (ATHLOS) consortium analysed eight longitudinal cohort studies from Asia, the South Pacific, Europe and North and South America to explore trajectories of healthy ageing. In the sample of individuals 45+ years, they found that cumulative disadvantage from low education and wealth may have increased the chances of poor health in early life, which then led to follow on effects persisting throughout older age and creating disparities in healthy ageing compared to more educated cohorts.^5^

Although the relationship between education and health outcomes is not fully understood, there are several theories about the mechanisms by which education has such a strong impact on health. The fundamental cause theory frames education as a fundamental cause of health and disease because it can determine whether an individual has a sufficient income, which can be used to access factors contributing to a healthy lifestyle including a healthy diet, means of staying physically active, being in a safe neighbourhood, etc. fundamental cause theory is corroborated by studies that have found that 30% of links between education and health can be attributed to economic factors that enable an individual to have a stable job and higher income, the latter of which can be used to make purchases that can be used to improve health.^3,6,7^

The human capital theory, meanwhile, frames education as a means to health rather than a fundamental cause of health.^6^ Human capital theory can be linked to the concept of ‘subjective motivational sets’, which are defined as ‘dispositions of evaluation, patterns of emotional reaction, personal loyalties, and various projects, as they might be called, embodying commitments of the agent’.^8^ Subjective motivational sets can be modified based on experience and knowledge, skills and reasoning and can be utilised by individuals to make choices to maintain and/or improve health. Human capital theory is corroborated by studies that have found that adults with less education are more likely to smoke, be physically inactive and have an unhealthy diet.^3^

The true causal mechanisms linking better education to better health likely involve a combination of both fundamental cause theory- and human capital theory-related factors.

## Intergenerational aspects of the education–health nexus

The Human Capital Model, which is tangentially linked to fundamental cause theory and human capital theory, addresses issues of intergenerational mobility. human capital model (HCM) posits that economic, biological and genetic factors play an important role in the transmission of health across generations because better educated parents invest more in their children’s human capital (i.e. skills, knowledge and health) and because some aspects of human capital can be transmitted biologically or genetically.^9^ Recent analyses of intergenerational and transgenerational data seem to support the human capital model.

Analyses of data from cohorts of parents at the juncture of a compulsory increase in schooling in Germany after World War II found causal effects of maternal schooling reducing rates of children’s smoking and overweight in adolescence with effects persisting into adulthood with lower rates of chronic conditions in these children.^10^ These results are corroborated by findings from the German Socio-Economic Panel study which consists of over 15,000 West German respondents born between 1925 and 1998. Children of lower educated parents were found to have worse physical health over the whole life course and worse mental health in mid-life and later life.^11^

Recent studies also suggest that epigenetic mechanisms could be associated with transmission of health, or disease, between generations. Epigenetic changes occur independent of changes to the DNA sequences of the genome (i.e. they do not involve DNA mutations) via modifications to DNA, histone proteins which DNA wraps around and/or the larger chromatin structure within the nucleus.^12^ At an intergenerational level, researchers have found children of Holocaust survivors, who have an increased risk of post-traumatic stress disorder and mood disorders, experience epigenetic changes within the hypothalamic–pituitary–adrenal axis.^1,12^ At a transgenerational level, undernutrition caused by famine in the Netherlands in World War II is thought to have led to epigenetic changes that contributed to increases in overweight and other health issues in the second generation as well as the grandchildren of those affected by the famine.^12^

This body of evidence points to an essential role for education to not only preserve health and wellbeing intra-generationally but also inter- and trans-generationally.

## Implications of the COVID-19 pandemic in the context of existing structural inequalities

The COVID-19 pandemic has led to the largest ever disruption to education systems globally, which was in the context of existing structural inequalities. Over 1.6 billion learners in over 190 countries (94% of the world’s student population) have been affected.^13–15^ Simulations suggest that ∼0.6 years of schooling will be lost globally due to school closures with higher levels for children from lower socioeconomic groups, who have been historically disadvantaged because of less access to high-quality education.^16^ It is further estimated that there will be an ∼25% increase in the proportion of children below minimum education proficiency and ∼24 million additional children and youth may drop out or not have access to school in 2021.^13–15,17^ Models from the World Bank suggest that five months-worth of school closures could lead to a loss of $10 trillion in working life earnings for students currently in school.^17^ In addition to the direct educational effects, school closures will also affect essential services and benefits provided to families in need, such as access to nutritious school meals, etc.^14,15^

## Leveraging the health-education nexus to build resilience

In light of the impact of health on education and the mechanistic underpinnings between education and health highlighted by fundamental cause theory, human capital theory and human capital model, it is clear that school disruptions caused by the COVID-19 pandemic can and will have significant negative intra-, inter- and trans-generational effects on health and wellbeing. From a strategic and operational perspective, however, it is difficult to exactly pinpoint who will be most negatively impacted, and when, because of the various internal and external factors that can influence an individual’s health trajectory.

Human beings can be viewed from the perspective of complex systems, which behave non-linearly and are not always predictable. A characteristic of complex systems is the presence of ‘tipping points’ – critical points at which ‘…the system is nearly unstable, with tiny disturbances possibly leading to global effects’.^18^ From a health and wellbeing perspective, being able to identify ‘tipping points’ would allow us to better understand when individuals might transition to poor health. Because of the complex nature of individuals, however, ‘tipping points’ cannot be defined *a priori* so we cannot predict exactly when individuals will reach a tipping point and transition to poor health.

This phenomenon is evident even in the studies cited in this manuscript – no factor has 100% penetrance in being able to cause health or poor health. This indicates that each individual will have a different level of stress they can tolerate before transitioning to a state of poor health.^3^ When and how transitions to states of poor health occur is a situation of fundamental uncertainty.^19^ In situations of fundamental uncertainty, the best approach to take for policies and practice is to focus on heuristics, simple rules of thumb that often outperform more complex data-informed models. In light of the outsize impact of education on current and future health, and the disruptions to education caused by the COVID-19 pandemic, the appropriate heuristic is to invest in education systems. Allocating more resources (financial and human) to support education systems is key to long-term health, wellbeing and resilience.^7,19^

Introducing education as an intervention is not, however, straightforward. In sociology, education is viewed as a means for creating as well constraining social opportunities, which can perpetuate inequalities. To avoid this, educational interventions need to be targeted at the level of social contexts at an early age to modify educational trajectories.^6^ Interventions can include identifying and working with the most vulnerable groups, strengthening school infrastructure, investing in teacher training and addressing existing curricular deficiencies.^17^ Furthermore, education needs to expand to include broader curricula on social and emotional health so that children have more awareness of how to stay healthy. These approaches are supported by several studies which demonstrate that intergenerational perpetuation of inequalities is lower in countries with greater educational public support programmes for low-income families with health and wellbeing benefits lasting into adulthood.^6,9,11,12^

## Conclusion

**Figure 1.**
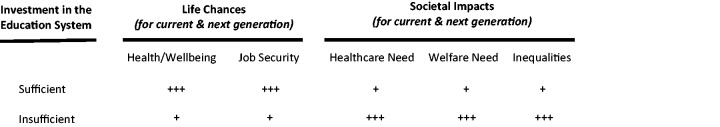
Impacts on life chances and society more generally from sufficient and insufficient levels of investment in the education system. Failing to address the COVID-19-related shocks to the education sector will entrench longer-term health inequalities across the life course and contribute to avoidable self-reinforcing cycles of poor health and wellbeing.^3,11^ It is a responsibility for policy-makers and healthcare professionals, as much as it is for education professionals, to act now to address these systemic vulnerabilities even if it requires moving away from the artificial silos we often find ourselves entrenched in.

If we had an inter-governmental Rawlsian ‘Veil of Ignorance’, where different government departments did not know which department they were part of and resource allocation decisions were made based on evidence rather than internal and external politics, the education sector would get a large chunk of resources given its potential for positive impact on health, wellbeing and the economy now and in the future as well as the outsized negative impacts that result from insufficient investment in this sector (Figure 1).^8^ We would likely see disinvestment from the healthcare sector and reallocation to education given the large burden of disease that could be avoided with better education systems. This is not, however, what we see. A recent analysis by the UK National Audit Office, found that the UK Education Sector received only ∼1% of COVID-19 support funding.^20^ It is a responsibility for policy-makers, healthcare professionals and education professionals to come together to address the systemic vulnerabilities we are exposing our society to by not investing enough in education.

## Declarations

**Ethics approval:** not applicable

**Guarantor:** AJ

**Contributorship:** AJ and CL contributed to the conceptualisation and writing of the manuscript. EH, SW, DG contributed to writing the manuscript.

**Provenance:** Not commissioned; peer reviewed by Olayiwola Ojo and Brian Birch.
